# Feasibility of an Individualized mHealth Nutrition (iNutrition) Intervention for Post-Discharged Gastric Cancer Patients Following Gastrectomy: A Randomized Controlled Pilot Trial

**DOI:** 10.3390/nu15081883

**Published:** 2023-04-13

**Authors:** Xiaohan Jiang, Jiamin Chen, Xiuhong Yuan, Yijia Lin, Yingliang Chen, Sijia Li, Qiuxiang Jiang, Hong Yu, Qianqian Du, Junsheng Peng

**Affiliations:** 1 School of Nursing, Sun Yat-sen University, Guangzhou 510006, China; 2 Department of Gastric Surgery, Department of General Surgery, Guangdong Provincial Key Laboratory of Colorectal and Pelvic Floor Diseases, The Sixth Affiliated Hospital, Sun Yat-sen University, Guangzhou 510006, China; 3 Department of Clinical Nutrition, The Sixth Affiliated Hospital, Sun Yat-sen University, Guangzhou 510006, China; 4 Department of Gastric Surgery, State Key Laboratory of Oncology in South China, Collaborative Innovation Center for Cancer Medicine, Sun Yat-sen University Cancer Center, Guangzhou 510006, China

**Keywords:** gastric cancer, mobile health, nutrition intervention, nutritional behavior, randomized controlled trial

## Abstract

(1) Background: A major challenge for post-discharged gastric cancer patients following gastrectomy is the impact of the anatomy change on decreased oral intake, nutritional status, and, ultimately, quality of life. The purpose of this study is to examine the feasibility and preliminary effects of an individualized mHealth nutrition (iNutrition) intervention in post-discharged gastric cancer patients following gastrectomy. (2) Methods: A mixed-method feasibility study with a parallel randomized controlled design was conducted. Patients were randomly assigned to either the iNutrition intervention group (*n* = 12) or the control group (*n* = 12). Participants completed measures at baseline (T0), four (T1), and twelve weeks (T2) post-randomization. (3) Results: Recruitment (33%) and retention (87.5%) rates along with high adherence and acceptability supported the feasibility of the iNutrition intervention for post-discharged gastric cancer patients following gastrectomy, echoed by the qualitative findings. The iNutrition intervention significantly improved participants’ nutritional behavior (*p* = 0.005), energy intake (*p* = 0.038), compliance with energy requirements (*p* = 0.006), and compliance with protein requirements (*p* = 0.008). (4) Conclusions: The iNutrition intervention is feasible and potentially benefits post-discharged gastric cancer patients following gastrectomy. A larger trial is required to establish the efficacy of this approach. Trial Registration: 19 October 2022 Chinese Clinical Trial Registry, ChiCTR2200064807.

## 1. Introduction

Gastric cancer is the fifth most common cancer in the world and the fourth major cause of cancer death [1]. Surgery is considered the only potentially curative treatment of gastric cancer [2]. A major challenge for postoperative gastric cancer patients is the impact of the anatomy change on decreased oral intake, nutritional status, and, ultimately, quality of life [3]. Approximately 80.4% of postoperative gastric cancer patients suffered from malnutrition [4]. Additionally, because of the normally short hospitalization periods, the presence of malnutrition is increasingly relocating to post-discharge settings [5]. After discharge, postoperative gastric cancer patients must adjust their eating habits to adapt to the anatomy change and minimize gastrointestinal symptoms (such as regurgitation, reflux, malabsorption, and dumping syndrome) to improve nutritional status and promote rehabilitation [3,6].

Clinical management guidelines recommend appropriate and effective nutritional therapy for postoperative gastric cancer patients to cope with the post-discharged nutritional challenge [7,8]. However, evidence-based interventions to support the implementation of these guidelines are lacking. Most previous studies and clinical practice have mainly focused on nutrition intervention during hospital stays [9,10]. There is lacking remote nutrition behavior interventions to help these patients transition from the medical center to the home [6,11].

**Figure 1:**
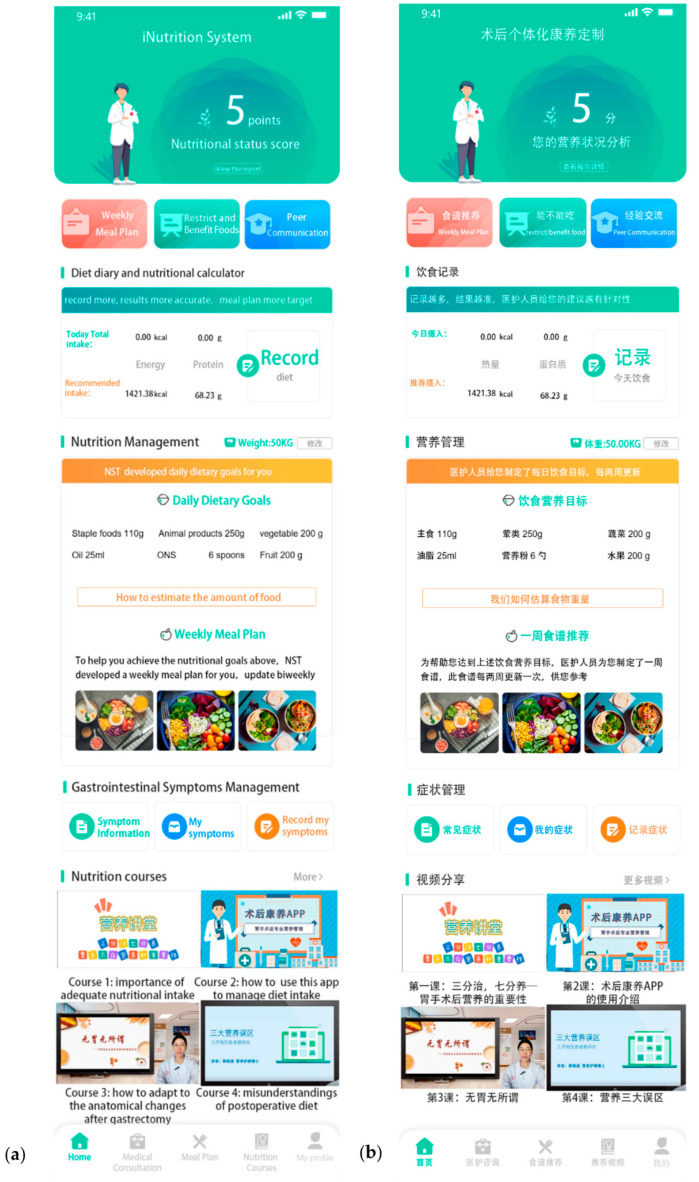
Screenshot of the iNutrition applet. (**a**) English version, (**b**) Chinese version.

Mobile health (mHealth) technology is an emerging platform for delivering remote behavioral interventions everywhere and at all times [12]. mHealth is defined as “medical and public health practice supported by mobile devices” [13]. New innovative mHealth tools for dietary intervention, such as the nutrition application (app), are gaining popularity [14]. Several studies have demonstrated that nutrition apps are effective in the improvement of diet behavior, diet intake, and weight management in populations with overweight, diabetes, and cancer survivors [15,16,17]. mHealth apps appear to be promising tools for implementing the transition towards healthier nutritional behaviors; however, they have not yet been tested for delivering nutrition intervention to post-discharged gastric cancer patients following gastrectomy [14,15]. In this study, we developed a WeChat applet called “iNutrition applet” to implement the intervention (Figure 1). WeChat applet is a mobile App based on the WeChat platform (Tencent Co., Ltd., Shenzhen, China) that is easily developed and does not require downloading, installation, or registration; therefore, it has been widely developed and used in mHealth intervention studies [18,19].

In this study, we developed the iNutrition intervention, which is the first mHealth app-based nutritional behavioral management program for post-discharged gastric cancer patients following gastrectomy. The aims of this study were to (1) examine feasibility (including recruitment, retention, adherence, and acceptability) of the iNutrition intervention; and (2) evaluate the preliminary effects of the iNutrition intervention on post-discharged gastric cancer patients following gastrectomy. We hypothesize that the iNutrition intervention is feasible. Despite the limited power of this exploratory study, the findings and participants’ feedback from this pilot study will inform the design for future fully-powered trials.

## 2. Materials and Methods

### 2.1. Design

This is a mixed-method feasibility study with a parallel randomized controlled design, comparing iNutrition intervention with usual care. The Medical Research Council (MRC) framework was used to guide the evaluation of the feasibility and optimize the study design [20]. This process aims to identify issues concerning recruitment, retention, acceptability, and adherence to the intervention. The present study was conducted in accordance with Good Clinical Practice (GCP) principles and was reported according to the guidelines of the Consolidated Standards of Reporting Trials (CONSORT) statement [21]. This trial was registered at the Chinese Clinical Trial Registry (ChiCTR2200064807).

### 2.2. Participants and Recruitment

A recruitment target of 12 patients per group was based on sample size recommendations for pilot studies by Julious et al. [22]. Similar sample sizes are seen in comparable pilot studies conducting nutrition intervention to cancer patients [23,24]. Participants were recruited from two participating university-affiliated tertiary-level hospitals (the Sixth Affiliated Hospital of Sun Yat-sen University and Affiliated Cancer Center of Sun Yat-sen University). Inclusion criteria included:

- histologically confirmed gastric adenocarcinoma;
- received D2 radical gastrectomy;
- access to broadband internet;
- patient’s age ≥ 18 years;
- patient agreed to participate in this trial through informed consent.

Exclusion criteria included: severe post-operative morbidity; evidence of active or recurrent disease including cardiovascular, respiratory, kidney, liver, or cerebrovascular diseases et al.; existence of other malignancies within last 5 years; patients had a diagnosed vision, hearing, or speech impairment.

Between December 2022 and January 2023, suitable participants (patients admitted with gastric cancer) were identified through screening of the patient databases of the participating hospitals. Once identified, we pre-contacted the potential participants to confirm eligibility in their process of diagnosis and treatment. Additionally, we then invited eligible patients to participate in the study one day before they discharged from the hospital. Written consent was obtained before attending the baseline visit.

### 2.3. Randomization and Blinding

Participants were randomly assigned to two groups after passing the screening visit and confirming their participation in the trial. A stratified randomization list (stratified by center) was generated using SPSS 26.0 software by a biostatistician who is independent of the investigator team, and allocations were concealed in sequentially numbered opaque envelopes. These envelopes were opened only after written consent had been obtained, ensuring that participants and research staff were blind prior to the allocation. Researchers who conducted the recruitment, data collection, and data analysis were blinded to group allocation.

### 2.4. Common Intervention for Both Groups

At each study site, all participants received usual care designated for discharged patients, in which a participant handbook outlining the benefits of adequate nutritional intake, a food atlas helping estimate the amount of food [25], and the time points for outcome assessments were given to all participants. All participants received nutrition education based on the handbook on the day of postoperative discharge. In addition, each participant of the intervention group and control group received 100 RMB (equal to 14 USD) to reimburse any costs (internet service, time, transport expense, etc.) related to completing measurements at baseline and follow-ups. Participants received 20 RMB when finishing the baseline assessment and received 40 RMB each time when finishing the T1 (4 weeks after discharged) and T2 (12 weeks after discharged) outcome assessments. All participants received a letter of appreciation from the project team upon completion of the study.

### 2.5. Specific Intervention (iNutrition Intervention)

#### 2.5.1. Intervention Details

Participants allocated to the intervention group were provided a 12-week individualized mHealth nutrition intervention (iNutrition intervention) aiming for optimal nutritional intake, using ordinary food and oral nutrition supplements (ONS), tailored to individual needs, preferences, and diet restriction. The intervention was led by a nutrition support team (NST) including administrative staff, nutritionists, surgeons, and nurses. An overview of the iNutrition intervention is provided in Figure 1. The iNutriton intervention will include (i) a WeChat mHealth applet called “iNutrition applet” (similar to a mHealth App) run via WeChat social media (Figure 1); and (ii) a biweekly nutrition consultation delivered through phone call.

(i) The iNutrition applet

The iNutrition applet consists of the following four modules (Figure 1).

a. Gastrointestinal symptoms management

We provide knowledge regarding the typical post-surgery gastrointestinal symptoms and symptom management guidelines after gastrectomy. Patients or caregivers could upload patients’ recent symptoms. The NST could give feedback according to patients’ recent symptoms in the biweekly nutrition consultation.

b. Nutrition management

There are four aspects: (1) nutritional status score, (2) nutritional requirement, (3) food diary with nutrition calculator, and (4) weekly meal plans along with recipes. Firstly, through the nutritional status score, participants can visually recognize their current nutritional status and changes in nutritional status through the Patient-Generated Subjective Global Assessment (PG-SGA). Secondly, the program could automatically calculate the nutritional requirements of participants after entering their weight and height. Additionally, if they modified weight in the program, the program would automatically update the nutritional requirement in real-time. Thirdly, patients or caregivers could keep a food diary through the program, and the nutrition calculator would calculate the energy and protein they took according to the Chinese Food Composition Tables in real-time [26]. Fourthly, we provided the concrete food requirements and a weekly meal plan which included the expected mealtime, the food items or ONS, and the amounts, as well as the preparation method reasonable for each dish, based on each patient’s dietary preferences, recovery of intestinal function following surgery, and nutritional requirements. In addition, we biweekly updated the content of each patients’ nutrition management module according to the feedback from telephone-delivered nutrition consultations.

c. Nutrition Knowledge

In the Nutrition Knowledge module, videos, texts, and images were used to display the latest nutritional knowledge regarding existing the guidelines, literature, and resources.

d. Communication center

In the communication center, participants were invited to share their experiences through texts, videos, and pictures as well as communicate with one another. Participants could also communicate with the NST and seek medical advice. The NST members read all messages every day and replied within 24 h.

(ii) Telephone-delivered Nutrition Consultation

The nutrition consultation was provided by a registered dietician (CJM) biweekly for 12 weeks. During each session of nutrition consultation, nutritional status and symptoms were recorded, and nutritional intake was assessed using tailored dietary interview strategies (incorporating 24 h recalls and qualitative information such as eating strategies and meal pattern). Then, the content of each patient’s iNutrition program was updated according to the participants’ nutritional status, nutritional intake, nutritional requirements, dietary challenges (such as gastrointestinal symptoms), recovery of intestinal function following gastrectomy, and dietary preferences through the telephone-delivered nutrition consultations. The target for participants was to optimize nutritional intake, ensuring adequate energy and protein intake, in accordance with the ESPEN guideline for postoperative cancer patients [7,8].

#### 2.5.2. Health Action Process Approach Theory

**Figure 2:**
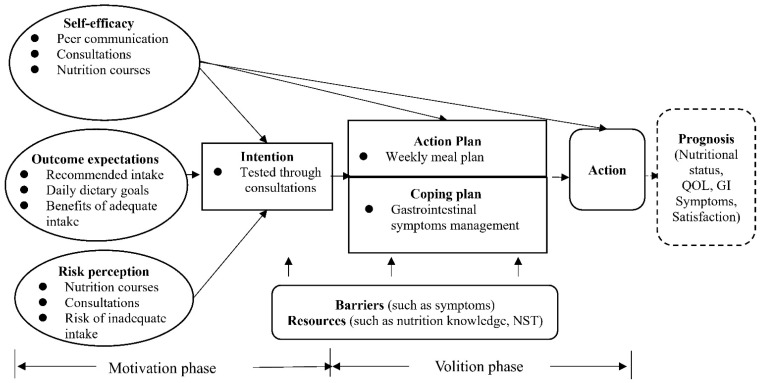
The Health Action Process Approach Theory.

**Figure 3:**
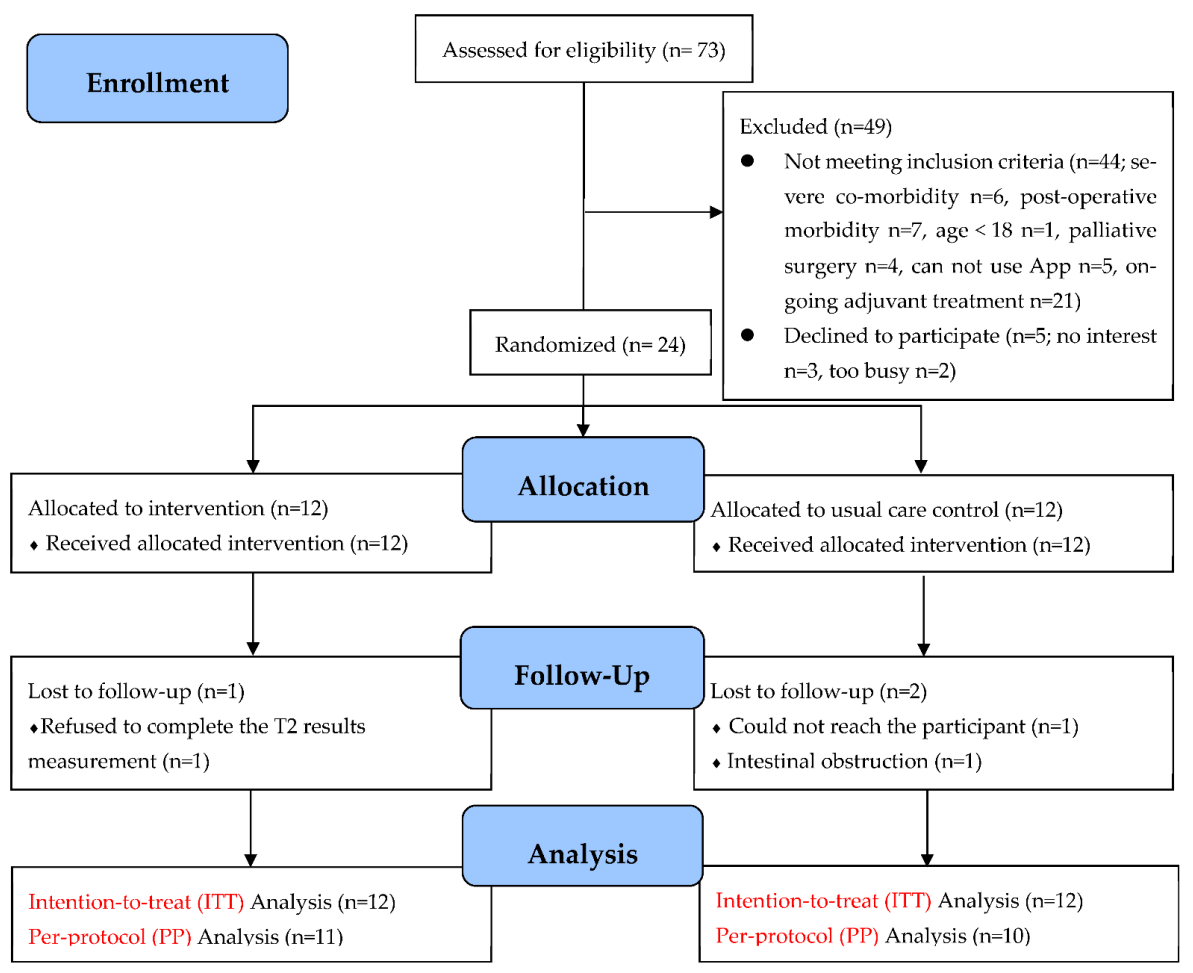
Enrollment, randomisation, and follow-up profile (CONSORT flow diagram).

The design of the iNutrition intervention is grounded in the Health Action Process Approach (HAPA) Theory [27], which characterizes the factors influencing health behaviors and addresses the intention–behavior gap of health behavior (Figure 2). According to the HAPA, changing into health behavior consists of two phases: (1) a motivation phase that includes self-efficacy, risk perceptions, and outcome expectancies that lead to a behavioral intention; and (2) a volition phase which occurs after a behavioral intention has been set in the motivation phase. The volition phase includes an action plan, coping plan, and self-efficacy, all of which lead to the actual health behavior. In addition, perceived barriers and resources play a crucial role in both phases. The design of the iNutrition intervention tried to address these key factors, as shown in Figure 3. Grounded in the HAPA theory, iNutrition aims to provide participants with better nutrition behavior during their recovery and help them to achieve adequate nutritional intake and nutritional status (Figure 2).

### 2.6. Outcomes

Outcome assessors were trained and masked to group allocation. Data were gathered upon hospital discharge (T0) and at 4 weeks (T1) and 12 weeks (T2) post-discharge.

#### 2.6.1. Quantitative Feasibility Measures

The primary outcome of this study was feasibility, which was defined by the criteria listed below.

- Recruitment rate: the percentage of the eligible study population who agree to participate.
- Retention rate: the percentage of enrolled participants who completed the post-intervention evaluation.
- Adherence of the intervention participants: (1) number of planned nutrition consultations completed and average duration of the consultations; (2) register rate—participants registered on the iNutrition applet and participants were allocated to the intervention group × 100%; (3) number of logins into iNutrition applet from baseline to post-test; (4) the percentage of days that participants were active on the iNutrition applet during the 12-week intervention period; (5) percentage of registered participants who visited each module of the iNutrition applet. Adherence was recorded through the analytics function of the iNutrition applet, and records of attended nutrition consultations were kept.
- Acceptability of the participants of the intervention arm: this was determined through the System Usability Scale (SUS) and the Net Promoter Score (NPS) findings. The SUS is a valid and reliable 10-item usability measurement scale designed to evaluate software products, such as websites and applets, and was graded on a 5-point Likert scale [28]. The NPS is a validated one-item questionnaire (“How likely would you be to recommend iNutrition applet to a friend?”) on a scale of 1 to 10, with 1 being the least-probable and 10 being the most-probable for recommending this applet to others. Respondents with scores ranging from 0 to 6 are considered detractors, those with 7 or 8 are considered passive, and those with 9 or 10 are considered promoters. To compute the total NPS score, we divided the percentage of detractors by the percentage of promoters, yielding a single number ranging from  −100% to +100%. Overall, a total NPS score greater than 0% indicated a stronger inclination to recommend the applet to others [29].

#### 2.6.2. Embedded Qualitative Feasibility Measures

To explore other aspects of acceptability, adherence, and potential improvements, we completed semi-structured interviews with the eleven patients of the intervention group. The interviews were audio-recorded and transcribed verbatim. All transcripts were coded by two researchers who were blind to the trial results (YC and HY). The inductive coding of all transcribed data was based on following the questions: ‘What is the experience of nutritional intake.’ ‘What is the experience of iNutrition intervention (including the applet and nutritional counselling)?’ ‘What are the barriers to and facilitators of postoperative adequate nutritional intake with the help of the iNutrition intervention?’ Each interview lasted approximately 20–30 min.

#### 2.6.3. Secondary Outcomes

The secondary aims were to investigate the impact of the iNutrition intervention on nutritional status, nutritional risk, nutritional intake, compliance with nutritional requirements, nutritional behavior, blood parameters, gastrointestinal symptoms, and quality of life (QOL). Secondary measurements were obtained at T0, T1, and T2 and included: weight; body mass index (BMI); nutritional status tested using PG-SGA points [30]; nutritional risk tested via Nutritional risk screening 2002 (NRS2002) [31]; nutritional intake tested using the food atlas-assisted 24 h recall method [25]; compliance with nutritional requirements tested using the proportion of patients’ nutritional intake that take up nutritional requirements (including energy and protein requirements) recommended by ESPEN guidelines [7,8]; nutritional behavior tested using HAPA scales for nutritional behavior [32]; gastrointestinal symptoms tested using gastrointestinal Symptom Rating Scale (GSRS) [33]; QOL tested using the Quality of Life Questionnaire-Core 30 (QLQ-C30) [34]. Secondary measures were merely exploratory, as the sample size was insufficient to demonstrate the treatment effect.

### 2.7. Statistical Analysis

Quantitative feasibility data: Statistical analysis was carried out through SPSS 26.0 (IBM Corp., Chicago, IL, USA). The analysis was performed using data from all patients recruited. We used descriptive statistics to assess study feasibility (recruitment rates, retention rates, adherence, and acceptability). Continuous data are summarized as the mean ± standard deviation (SD). Additionally, categorical data are presented as percentage (%).

Secondary outcomes: To summarize the characteristics of the participants, descriptive statistics were utilized. To examine baseline differences across groups, the Chi-square test, Mann–Whitney U test, and independent *t*-test were used. Generalized estimating equations (GEE) with Bonferroni correction were used to examine changes in clinical outcomes across time (T0–T1–T2) and between-group comparisons. The GEE analyses were carried out in two ways: by protocol (only those who completed the protocol were included: iNutrition group *n* = 11; control group *n* = 10), and by intention-to-treat (including those who did not complete the study: iNutrition group *n* = 12; control group *n* = 12) using the last-observation-carried-forward method. A *p* < 0.05 value was regarded as statistically significant. Cohen’s d was used to report effect sizes (≥0.2 = small; ≥0.5 = moderate; ≥0.8 = large).

Qualitative feasibility data: NVivo software (Version 11, QRS International) was used to analyze the qualitative data. The framework technique was utilized in the qualitative analysis, which includes systematic and interrelated steps of filtering and charting coded qualitative data, followed by mapping patterns in a search for understanding and explanation. The pre-existing framework, the Health Action Process Approach (HAPA) theory, was implemented to code data. Instead of constructing themes from the data, the interviews were coded by utilizing components of the HAPA model constructs as themes. Overarching patterns that demonstrated acceptability of the iNutrition intervention and factors impacting adherence/non-adherence to the iNutrition intervention were identified.

## 3. Results

### 3.1. Baseline Characteristics

The study involved 24 participants distributed into two groups: the intervention group (*n* = 12) and control group (*n* = 12). The participants’ age ranged from 36 to 72 years (mean ± SD, 54.88 ± 10.07) and 66.7% were men (*n* = 16). BMI ranged from 16.22 to 31.22 kg/m^2^ and the average BMI was 23.55 ± 3.34 kg/m^2^. There were no significant differences in demographic or baseline outcomes between the two groups (Table 1).

### 3.2. Recruitment and Retention

A flow chart of recruitment and retention is presented in Figure 3. All of the gastric cancer patients in the two study sites were assessed for eligibility from December 2022 to January 2023. Of the seventy-three patients assessed for eligibility, forty-four (60%) were not eligible and five (7%) declined. We successfully recruited 24 post-discharged gastric cancer patients following gastrectomy over a four-week period. The recruitment rate was 33% (24/73). Of the 24 patients recruited (intervention *n* = 12, control *n* = 12), 21 (intervention *n* = 11, control *n* = 10) completed the study, resulting in an 87.5% retention rate: one participant of the intervention group was lost to follow-up because of refusing to complete the results measurement at T2, and two participants in the control group were lost to follow-up because either they could not be reached (*n* = 1) or because of intestinal obstruction (*n* = 1).

### 3.3. Adherence and Acceptability

#### 3.3.1. Adherence

Results of adherence are from participants who completed the iNutrition intervention (*n* = 12) (Table 2). The mean number of the actually completed sessions of nutrition consultations by each participant was 5.33 ± 0.78 sessions (88.89% ± 12.98%). The average duration of the nutrition consultations was 23.60 (SD = 8.94) minutes.

All participants were registered onto the iNutrition applet. Participants averagely used 3.42 ± 0.79 modules of the four modules of the applet. Over 58.33% of participants still visited the applet at post-test. On the iNutrition applet, participants were active a median of 89 (range: 37–1975) times in 56.5 (range: 10–84) days during the 12-week intervention. The mean percent of days within the 12-week-intervention period that participants logged into the iNutrition applet was 64.88% (SD 28.04%).

#### 3.3.2. System Usability Scale

The total score of the 11 patients who completed the SUS was 77.27 out of 100 (SD = 10.69). Results from each item of the SUS are presented in Table 3.

#### 3.3.3. Net Promoter Score

The NPS was used to assess participants’ acceptability of the iNutrition applet and the intervention. The NPS was completed by 11 participants in the intervention arm. The overall NPS for the patient cohort was positive, at 18.2% (with 36.4% reporting as promoters, 45.5% as passive, and 18.2% as detractors).

### 3.4. Qualitative Feasibility Data

An end-of-trial interview was conducted with eleven patients in the intervention group. The factors influencing acceptability and adherence to the iNutrition intervention (based on the HAPA theory) are reported in the Supplementary Material S2.

In summary, to adhere with the iNutrition intervention, patients had to perceive nutritional risk, realize the importance of nutrition intake, and expect an improvement in physical and psychological status after the intervention. Participants reported the iNutrition intervention was enjoyable and restorative. However, only some of these patients reported improved nutritional intake, weight, or quality of life. This may be due to some barriers and resources influencing participants’ adherence to the intervention. No improvement in physical condition (such as weight), gastrointestinal symptoms, difficulties with the technology, or conflict knowledge prevented the participants’ adherence. The multidisciplinary nature of the program, the biweekly nutrition consultation through phone calls, the evidence-based knowledge in the applet, and the concrete action and coping plan, could all have motivated adherence. Family members and caregivers also had an impact on the capacity and willingness to adhere to the iNutrition intervention.

### 3.5. Secondary Measures

In the intention-to-treat (ITT) analysis, the iNutrition intervention had significant group-by-time interaction effects on energy intake (Wald χ^2^ = 6.54, *p* = 0.038), compliance with energy requirements (Wald χ^2^ = 10.28, *p* = 0.006), compliance with protein requirements (Wald χ^2^ = 9.57, *p* = 0.008), and nutritional behavior (Wald χ^2^ = 10.52, *p* = 0.005) over time (Table 4). However, overall changes in protein intake were nonsignificant (Wald χ^2^ = 4.85; *p* = 0.088). Improvements in protein intake occurred at T2 (β = 15.77, 95% CI [0.26, 31.27], *p* = 0.046), but they were not significant at T1 (β = 5.76, 95% CI [−13.95, 25.47], *p* = 0.567) (Table 4).

**Figure 4:**
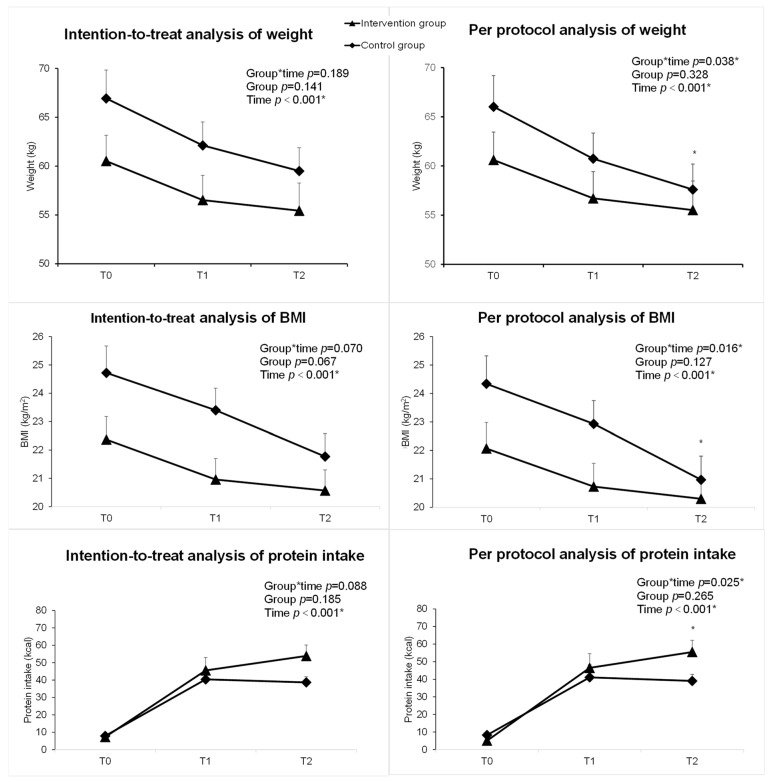
GEE analysis of weight, BMI, and protein intake in the intervention and control groups of the trial. The left panel shows the intention-to-treat analysis. The right panel shows the per-protocol analysis. (* = *p <* 0.05, triangle represents intervention group, rectangle represents control group).

The sensitivity analysis, which compared the ITT analysis (*n* = 24) and Per-protocol (PP) approach (*n* = 21, study completers), is in the Supplementary Material S1. In the ITT analysis, there was a largely nonsignificant change in the weight (*p* = 0.189), BMI (*p* = 0.070) and protein intake (*p* = 0.088) over time (Figure 4). However, in the PP analysis, the reductions in weight (*p* = 0.038) and BMI (*p* = 0.016) were lower in the intervention group compared to the control group. Additionally, the improvement of protein intake (*p* = 0.025) was greater in patients in the intervention group compared to the control group (Figure 4).

There was no significant difference between the intervention and control groups over time regarding changes in PG-SGA (ITT analysis: Wald χ^2^ = 0.99; *p* = 0.609; PP analysis: Wald χ^2^ = 1.45; *p* = 0.484), NRS2002 (ITT analysis: Wald χ^2^ = 2.39; *p* = 0.303; PP analysis: Wald χ^2^ = 2.65; *p* = 0.266), GSRS (ITT analysis: Wald χ^2^ = 1.02; *p* = 0.601; PP analysis: Wald χ^2^ = 4.03; *p* = 0.133), and QOL scores (ITT analysis: Wald χ^2^ = 0.73; *p* = 0.695; PP analysis: Wald χ^2^ = 0.54; *p* = 0.764) in both the ITT analysis (Table 4) and PP analysis (Supplementary Material S1).

## 4. Discussion

This randomized-controlled pilot trial assessed the feasibility and preliminary effects of the iNutrition intervention for post-discharged gastric cancer patients following gastrectomy. Despite being affected by the COVID-19 pandemic, recruitment to the trial was in line with pre-study expectations as described in the clinical trial registration (ChiCTR2200064807). A high retention rate was attained, indicating that online intervention and surveys were feasible methods. Adherence and acceptability of the iNutrition intervention were also high, with most participants completing the intervention components and receiving high average scores across acceptability measures (NPS and SUS), echoed by our qualitative findings. Evidence from this study suggests that the iNutrition intervention is a feasible approach that is highly acceptable to post-discharged gastric cancer patients following gastrectomy. The trial was not structured to evaluate the effects on nutritional status, nutritional risk, weight, BMI, protein intake, symptoms, and quality of life, but encouraging changes in nutritional behavior, energy intake, and compliance with energy and protein requirements were observed. There is a strong belief that the iNutrition intervention should be an optional therapy for post-discharged gastric cancer patients following gastrectomy. Overall, the positive findings provide support for further development and evaluation of the iNutrition intervention.

The current study successfully recruited 24 post-discharged gastric cancer patients following gastrectomy over a four-week period. The recruitment rate of 33% was similar with other mHealth interventions [23,35]. A high exclusion or refusal rate has been observed in other multicomponent mHealth interventions in cancer care [23,35]. In this study, the main reason for the low recruitment rate was that in order to establish a relationship of trust with the potential participants in order to improve the retention rate, the nutrition support team (NST) pre-contacted and screened the potential participants when they were admitted to the hospital [36]. Additionally, the majority of these potential participants were excluded in the process of diagnosis and treatment during hospital stays because of not meeting the inclusion criteria due to severe co-morbidity, inoperable, adjuvant treatment, and post-operative morbidity, for instance. Although the recruitment rate was relatively low, the recruitment was still considered successful overall based on the total number of participants recruited within the pre-determined period of time.

The retention rate achieved in the current study was good, with 87.5% reporting complete data during the 12-week follow-up. In comparation, similar studies providing nutrition interventions through a combination of phone calls and apps have similar retention rates of between 75–90.7% after the 12-week intervention [23,37,38]. The current study’s high retention rate was attained with minimal reimbursements, including 100 RMB (equivalent to 14 USD) for completing measures at baseline and follow-up. The high retention rate may benefit from the trust relationship between the intervenors and the participants through the pre-contact in the hospital and different methods of communication (messages and telephone calls) used to remind participants to complete the assessments [39,40].

In general, patients were highly involved with the iNutrition intervention, in terms of attendance and high acceptability scores of the NPS and SUS, echoed by the qualitative findings. In this study, participants completed 88.89% ± 12.98% planned nutrition consultations and used the iNutrition applet a median of 89 (range: 37–1975) times of during the 12 weeks of the intervention, suggesting a high adherence among participants. Biweekly telephone-delivered nutrition consultations implemented to improve adherence may have had a role in prompting participation, as suggested by interviews. Although adherence with the mHealth intervention is difficult to quantify [41], the current study’s level of adherence was high because the majority of participants completed the key intervention components (nutrition consultation and usage of the iNutrition applet). Usability results from the SUS indicate that the iNutrition applet was simple to use and that patients could follow the iNutrition protocols without assistance. Additionally, the mean score of NPS of participants was 18.2%, implying overall satisfaction with the app. These findings suggest that the iNutrition model was convenient, improved access to healthcare, and provided high-quality healthcare interaction. The qualitative data revealed some mixed results in terms of participants’ acceptability and adherence. Participants appreciated the convenience of the iNutrition intervention. A fundamental strength of the mHealth approach is the removal of travel-related barriers to postoperative nutrition interventions [42]. However, changes in physical condition (such as weight), difficulties with the technology, gastrointestinal symptoms, conflict knowledge or evidence-based knowledge, multidisciplinary support, nutrition consultation, and the concrete plan, could all have influenced participants’ adherence. The qualitative findings demonstrated the intervention’s positive impact and suggest continuation with a larger trial is worthwhile while also helping to refine aspects of the trial design.

Furthermore, preliminary evidence of outcome improvements associated with the iNutrition intervention suggests that further testing in a sufficiently powered study is warranted. In the ITT analysis, including all randomized participants who started the trial, there was a significant between-group improvement in nutritional behavior, energy intake, and compliance with energy and protein requirements in the intervention participants compared with the control participants. Changes in protein intake, weight, BMI, nutritional status, nutritional risk, gastrointestinal symptoms, and quality of life were non-significant, however much they moved in the desired direction. Comparatively, in a nutrition intervention among perioperative upper gastrointestinal cancer patients, there was no significant effect on weight, nutritional status, and quality of life following 18 weeks of telephone or electronic nutrition counselling [43]. In a 6-month postoperative phone-based dietary intervention, there was no improvement in dietary intake, nutritional status, and quality of life for people having had major upper gastrointestinal surgery [44]. The current study results are promising in comparison with these studies due to the benefits of a tailored mHealth intervention approach made up of synchronous (telephone-based nutrition consultation) and asynchronous (applet usage) methods. However, the sample size of this study was insufficient. Weight, BMI, and protein intake were borderline significant in the PP analysis while they were non-significant in the ITT analysis, indicating the potential positive impact of the iNutrition intervention on these outcomes. The important question of whether the iNutrition program may translate into an improvement in weight, BMI, and protein intake needs to be tested in a large-scale and well-powered study.

### Strengths and Limitations

This study provides useful information in relation to the feasibility and impact of the iNutrition intervention for post-discharged gastric cancer patients following gastrectomy, thus addressing an existing gap in the literature. The strengths of this study include the RCT design, combination of quantitative and qualitative measures, tailored mHealth app, and excellent retention rate. Additionally, a theory-based approach was applied for intervention development. Additionally, the MRC framework was used to ensure a complete feasibility evaluation. Although the results of this intervention are encouraging, there are several limitations that warrant consideration. Firstly, the small sample size in this study was chosen to be appropriate for the primary outcome (feasibility), but it was insufficient to enable powerful statistical analysis of secondary measures in this study. As a result, the secondary measures should be considered preliminary and treated with caution. Secondly, because the follow-up was restricted to post-intervention, the long-term consequences of the intervention in this population are unknown. Thirdly, we pre-contacted and screened patients when they were admitted to the hospital to establish a relationship of trust with the potential participants, which may have improved the retention rate. The participants received reimbursement for completing measurements at baseline and follow-ups, which may also have improved the retention rate. Fourthly, because of the pilot nature of this study, potential confounding factors such as supplementary pharmacy use and physical activity were not considered in the assessment of nutritional status or other health outcomes. These factors will be considered in the design and analyses of the future trial.

## 5. Conclusions

The iNutrition intervention is the first individually tailored, symptom-directed and theory-based nutritional behavioral management program developed and tested in post-discharged gastric cancer patients following gastrectomy. Our results showed that the iNutrition intervention is feasible and has potential benefits in terms of nutritional behavior, energy intake, and compliance with energy and protein requirements. Future studies with a more rigorous design and larger sample size are needed to establish efficacy in post-discharged gastric cancer patients following gastrectomy and those with other types of cancers.

## Figures and Tables

**Table 1:** Demographic, clinical data, and outcomes at baseline.

Characteristics	Intervention (*n* = 12)	Control (*n* = 12)	*p*-Value
Gender, *n* (%)			1.00 ^b^
Male	8 (66.7%)	8 (66.7%)	
Female	4 (33.3%)	4 (33.3%)	
Age (years), mean (SD)	54.08 (10.54)	55.67 (9.98)	0.78 ^a^
Education (years)	14.17 ± 2.76	12.42 ± 3.73	0.76 ^c^
Work situation, *n* (%)			1.00 ^b^
Employed	6 (50%)	5 (41.7%)	
Unemployed	2 (16.7%)	3 (25%)	
Retired	4 (33.3%)	4 (33.3%)	
Chronic illnesses, *n* (%)			1.00 ^b^
0	3 (25%)	2 (16.7%)	
1–2	5 (41.7%)	6 (50%)	
3 or above	4 (33.3%)	4 (33.3%)	
Tumor location			0.86 ^b^
Proximal	2 (16.7%)	2 (16.7%)	
Middle	3 (25%)	5 (41.7%)	
Distal	7 (58.3%)	5 (41.7%)	
Pathological stage			0.15 ^b^
I	2 (16.7%)	3 (25%)	
II	7 (58.3%)	2 (16.7%)	
III	2 (16.7%)	6 (50%)	
IV	1 (8.3%)	1 (8.3%)	
Resection extended			1.00 ^b^
Partial gastrectomy	9 (75%)	9 (75%)	
Total gastrectomy	3 (25%)	3 (25%)	
Whether received Neoadjuvant treatment before the surgery			1.00 ^b^
Yes	4 (33.3%)	4 (33.3%)	
No	8 (66.7%)	8 (66.7%)	
Baseline secondary outcomes (mean, SD)			
PG-SGA	6.67 (2.23)	7.58 (2.02)	0.30 ^a^
NRS2002	4.92 (0.67)	4.83 (0.39)	0.59 ^c^
Weight	60.52 (10.52)	65.02 (9.85)	0.29 ^a^
BMI	22.37 (3.43)	24.72 (2.93)	0.09 ^a^
Energy intake	225.07 (150.44)	249.01 (100.34)	0.20 ^c^
Protein intake	7.28 (7.96)	7.90 (5.38)	0.35 ^c^
Compliance with energy requirements	0.15 (0.10)	0.15 (0.06)	0.44 ^c^
Compliance with protein requirement	0.10 (0.11)	0.10 (0.07)	0.59 ^c^
HAPA Scale	3.73 (0.42)	3.51 (0.29)	0.14 ^a^
GSRS	6.58 (3.58)	7.25 (3.47)	0.65 ^a^
QLQ-C30	78.60 (14.45)	72.35 (12.29)	0.27 ^a^

Note: SD = standard deviation; PG-SGA = Patient-Generated Subjective Global Assessment; NRS2002 = Nutritional risk screening 2002; HAPA Scale = Health Action Process Approach Theory Scale; GSRS = Gastrointestinal Symptom Rating Scale; QLQ-C30 = Quality of Life Questionnaire-Core 30. ^a^ Tested with Unpaired *t*-test; ^b^ Chi-square tests, ^c^ Mann–Whitney U test.

**Table 2:** Adherence to different program components (*n* = 12).

Program Component	
Telephone-delivered nutrition consultation	
Attendance rate of nutrition consultation (mean ± SD)	88.89% ± 12.98%
Average duration of the nutrition consultations	23.60 ± 8.94 min
iNutrition applet	
Register rate (until T2), *n* (%)	100 (100%)
Usage at T1, yes, *n* (%)	11 (91.7%)
Usage at T2, yes, *n* (%)	7 (58.33%)
Logins into the applet (T0-T2), Mdn (IQR)	89 (98.25)
The number of days active on the applet, Mdn (IQR)	56.5 (20)
Percentage of days active on the applet (T0-T2), Mean (SD)	64.88% ± 28.04%
Visits per module, *n* (%)	
Module 1: Nutrition management	12 (100%)
Module 2: Gastrointestinal symptoms management	9 (75%)
Module 3: Nutrition Knowledge	10 (83.33%)
Module 4: Communication center	10 (83.33%)

Note: T0 = at hospital discharge, T1 = 4 weeks after discharge, T2 = 12 weeks after discharge.

**Table 3:** System Usability Scale (*n* = 11).

Items	Mean Score (SD), Max = 5
1. I think that I would like to use this system frequently	4.36 ± 0.67
2. I found the system unnecessarily complex	2.27 ± 0.65
3. I thought the system was easy to use	4.36 ± 0.51
4. I think that I would need the support of a technical person to be able to use this system	2.09 ± 1.14
5. I found the various functions in this system were well integrated	3.82 ± 0.75
6. I thought there was too much inconsistency in this system	1.91 ± 0.75
7. I would imagine that most people would learn to use this system very quickly	4 ± 0.63
8. I found the system very cumbersome to use	1.73 ± 0.65
9. I felt very confident using the system	4.27 ± 0.47
10. I needed to learn a lot of things before I could get going with this system.	1.91 ± 0.70

**Table 4:** Generalized Estimating Equations (GEE); results of the comparison of secondary outcomes between the intervention and control groups in the intention-to-treat (ITT) analysis.

Measures		Intervention Group (*n* = 12)	Control Group (*n* = 12)	Group-by-Time Interaction Effects			Effect Size T0-T1 T1-T2
		Mean (SE)	Mean (SE)	Wald χ^2^	β (95% CI)	*p*	d
PG-SGA	T0	6.67 (0.62)	7.58 (0.56)	0.99			
	T1	7.83 (0.73)	9.08 (0.79)	(*p* = 0.609)	−0.33 (−3.06, 2.39)	0.811	0.10
	T2	5.50 (0.85)	7.50 (0.86)		−1.08 (−3.50, 1.33)	0.379	0.38
NRS2002	T0	4.92 (0.19)	4.83 (0.11)	2.39			
	T1	3.33 (0.30)	3.50 (0.28)	(*p* = 0.303)	−0.25 (−1.17, 0.67)	0.593	0.23
	T2	2.75 (0.32)	3.33 (0.25)		−0.67 (−1.60, 0.27)	0.162	0.60
Weight	T0	60.52 (2.91)	66.93 (2.64)	3.33			
	T1	56.52 (2.40)	62.12 (2.53)	(*p* = 0.189)	0.82 (−1.48, 3.11)	0.485	0.07
	T2	55.43 (2.38)	59.50 (2.84)		2.34 (−0.40, 5.08)	0.094	0.78
BMI	T0	22.37 (0.95)	24.72 (0.81)	5.31			
	T1	20.96 (0.78)	23.40 (0.74)	(*p* = 0.070)	−0.09 (−1.18, 0.99)	0.868	0.07
	T2	20.57 (0.81)	21.77 (0.73)		1.15 (−0.07, 2.38)	0.066	0.78
Energy intake	T0	225.07 (41.58)	249.01 (27.73)	6.54			
	T1	991.52 (115.78)	770.00 (58.22)	(*p* = 0.038 *)	245.47 (−18.18, 509.11)	0.068	0.78
	T2	1072.52 (86.94)	811.72 (56.64)		284.74 (65.31, 504.17)	0.011 *	1.08
Protein intake	T0	7.28 (2.20)	7.90 (1.49)	4.85			
	T1	45.55 (7.41)	40.40 (6.02)	(*p* = 0.088)	5.76 (−13.95, 25.47)	0.567	0.24
	T2	53.83 (6.33)	38.68 (3.20)		15.77 (0.26, 31.27)	0.046 *	0.85
Compliance with energy requirement	T0	0.15 (0.03)	0.15 (0.02)	10.28			
	T1	0.67 (0.07)	0.51 (0.04)	(*p* = 0.006 *)	0.17 (0.00, 0.33)	0.046 *	0.85
	T2	0.73 (0.05)	0.53 (0.03)		0.20 (0.08, 0.33)	0.001 *	1.27
Compliance with protein requirement	T0	0.10 (0.03)	0.10 (0.02)	9.57			
	T1	0.63 (0.09)	0.56 (0.09)	(*p* = 0.008 *)	0.08 (−0.18, 0.34)	0.563	0.25
	T2	0.82 (0.08)	0.52 (0.04)		0.30 (0.09, 0.50)	0.004 *	1.19
HAPA	T0	3.73 (0.11)	3.50 (0.08)	10.52			
	T1	3.92 (0.08)	3.03 (0.12)	(*p* = 0.005 *)	0.67 (0.26, 1.07)	0.001 *	1.39
	T2	3.94 (0.12)	3.08 (0.13)		0.64 (0.17, 1.12)	0.008 *	1.12
GSRS	T0	6.58 (0.99)	7.25 (0.96)	1.02			
	T1	6.33 (1.46)	9.17 (1.30)	(*p* = 0.601)	−2.17 (−6.74, 2.41)	0.353	0.40
	T2	6.50 (1.41)	8.92 (1.41)		−1.75 (−5.68, 2.18)	0.382	0.37
QoL	T0	78.60 (3.99)	72.35 (3.40)	0.73			
	T1	77.08 (3.77)	66.86 (3.46)	(*p* = 0.695)	3.97 (−6.29, 14.23)	0.448	0.32
	T2	75.61 (3.45)	68.48 (3.09)		0.89 (−8.12, 9.90)	0.847	0.08

Note: * = *p <* 0.05; SD = standard deviation; PG-SGA = Patient-Generated Subjective Global Assessment; NRS2002 = Nutritional risk screening 2002; HAPA Scale = Health Action Process Approach Theory Scale; GSRS = Gastrointestinal Symptom Rating Scale; QLQ-C30 = Quality of Life Questionnaire-Core 30.

## Data Availability

The datasets used and/or analyzed during the current study are available from the corresponding author on reasonable request.

## Supplementary Materials

The following supporting information can be downloaded at: https://www.mdpi.com/article/10.3390/nu15081883/s1, File S1: Sensitivity analysis of the secondary outcomes; File S2: Qualitative analysis.
