# Supplementary material for: Feasibility of an Individualized mHealth Nutrition (iNutrition) Intervention for Post-Discharged Gastric Cancer Patients Following Gastrectomy: A Randomized Controlled Pilot Trial
Source: Nutrients. 2023 Apr 13;15(8):1883. doi: 10.3390/nu15081883 (PMC10144256; doi:10.3390/nu15081883)
Supplement: Supplementary file 1 [file nutrients-15-01883-s001.zip › Supplementary material 1.pdf]

Supplementary material 1 Sensitivity analysis of the secondary outcomes

Table S1. GEE results of the secondary outcomes between the intervention and control groups in the per-protocol analysis.

Measures		Intervention group (n=11)	Control group (n=10)	Group effect	Time effect	Group-by-time interaction effects			Effect size T0-T1 T1-T2
		Mean (SE)	Mean (SE)	Wald $\chi2$	Wald $\chi2$	Wald $\chi2$	β (95% CI)	P	d
PG-SGA	T0	6.82 (0.65)	7.90 (0.61)	6.07	14.30	1.45			
	T1	7.91 (0.80)	9.90 (0.64)	$(P=0.014^*)$	$(P=0.001^*)$	$(P=0.484)$	-0.91 (-3.76, 1.95)	0.533	-0.28
	T2	5.36 (0.92)	8.00 (0.92)				-1.56 (-4.11, 1.01)	0.234	-0.55
NRS2002	T0	5.09 (0.09)	4.90 (0.10)	0.17	92.97	2.65			
	T1	3.36 (0.32)	3.40 (0.29)	$(P=0.677)$	$(P < 0.001^*)$	$(P=0.266)$	-0.23 (-1.12, 0.66)	0.616	-0.23
	T2	2.73 (0.34)	3.20 (0.24)				-0.66 (-1.51, 0.18)	0.123	-0.70
Weight	T0	60.61 (3.17)	66.02 (2.84)	0.96	102.66	6.53			
	T1	56.71 (2.61)	60.74 (2.72)	$(P=0.328)$	$(P < 0.001^*)$	$(P=0.038^*)$	1.38 (-0.98, 3.74)	0.252	0.51
	T2	55.52 (2.60)	57.60 (2.95)				3.33 (0.69, 5.97)	0.013^*	1.10
BMI	T0	22.07 (0.98)	24.34 (0.91)	2.32	65.26	8.23			
	T1	20.73 (0.82)	22.93 (0.81)	$(P=0.127)$	$(P < 0.001^*)$	$(P=0.016^*)$	0.07 (-1.14, 1.28)	0.910	0.05
	T2	20.30 (0.83)	20.97 (0.61)				1.61 (0.36, 2.85)	0.012^*	1.16
Energy intake	T0	185.62 (18.97)	243.61 (33.02)	3.90	187.45	10.33			
	T1	1002.04 (125.83)	753.60 (65.96)	$(P=0.048^*)$	$(P < 0.001^*)$	$(P=0.006^*)$	306.44 (35.15, 577.73)	0.027^*	0.99
	T2	1090.40 (92.99)	803.66 (64.90)				344.74 (131.59, 557.88)	0.002^*	1.44

Protein intake	T0	5.08 (0.69)	8.28 (1.76)	1.24	109.83	7.38			
	T1	46.48 (8.03)	41.10 (7.15)	(P=0.265)	(P < 0.001*)	(P=0.025*)	8.58 (-12.68, 29.83)	0.429	0.36
	T2	55.52 (6.67)	39.04 (3.72)				19.68 (4.47, 34.88)	0.011*	1.14
Compliance with energy requirement	T0	0.12 (0.01)	0.15 (0.02)	4.88	280.13	14.48			
	T1	0.67 (0.08)	0.51 (0.05)	(P=0.027*)	(P < 0.001*)	(P=0.001*)	0.19 (0.02, 0.37)	0.030	1.00
	T2	0.74 (0.06)	0.54 (0.04)				0.23 (0.11, 0.35)	< 0.001*	1.63
Compliance with protein requirement	T0	0.07 (0.01)	0.11 (0.02)	2.29	151.55	13.78			
	T1	0.64 (0.09)	0.58 (0.11)	(P=0.130)	(P < 0.001*)	(P=0.001*)	0.10 (-0.18, 0.38,)	0.493	0.31
	T2	0.84 (0.09)	0.54 (0.04)				0.34 (0.15, 0.54)	< 0.001*	1.53
HAPA Scale	T0	3.76 (0.12)	3.55 (0.09)	34.81	3.67	10.04			
	T1	3.91 (0.08)	2.98 (0.14)	(P < 0.001*)	(P=0.160)	(P=0.007*)	0.72 (0.28, 1.17)	0.002*	0.68
	T2	3.94 (0.13)	3.04 (0.15)				0.69 (0.16, 1.22)	0.011*	1.18
GSRS	T0	7.00 (0.99)	6.70 (0.98)	2.00	2.92	4.03			
	T1	6.64 (1.56)	10.00 (1.30)	(P=0.158)	(P=0.232)	(P=0.133)	-3.66 (-8.15, 0.82)	0.109	-0.74
	T2	6.82 (1.50)	9.70 (1.48)				-3.18 (-6.76, 0.39)	0.081	-0.79
QLQ-C30	T0	80.48 (3.89)	71.09 (3.95)	4.95	4.18	0.54			
	T1	77.26 (4.11)	65.11 (3.71)	(P=0.052)	(P=0.124)	(P=0.764)	2.76 (-7.91, 13.43)	0.612	0.23
	T2	75.66 (3.77)	67.05 (3.30)				-0.78 (-9.98, 8.41)	0.868	0.07

Notes. GEE = Generalized estimating equations, T0 = baseline; T1 = middle of the intervention; T2 = immediately after the intervention; PG-SGA = Patient-Generated Subjective Global Assessment; NRS2002 = Nutritional risk screening 2002; HAPA Scale= Health Action Process Approach Theory Scale; GSRS = Gastrointestinal Symptom Rating Scale; QLQ-C30 = European Organization for the Research and Treatment of Cancer Quality of Life Questionnaire.

Table S2. GEE results of the comparison between ITT and per-protocol analyses

Outcome Measure	GEE Analysis	ITT Analysis		Per protocol analysis	
		Wald χ2	p-value	Wald χ2	p-value
PG-SGA	Group effect	3.25	0.072	6.07	0.014*
	Time effect	13.18	< 0.001*	14.30	0.001*
	Group x time	0.99	0.609	1.45	0.484
NRS2002	Group effect	0.84	0.361	0.17	0.677
	Time effect	60.42	< 0.001*	92.97	< 0.001*
	Group x time	2.39	0.303	2.65	0.266
Weight	Group effect	2.17	0.141	0.96	0.328
	Time effect	82.75	< 0.001*	102.66	< 0.001*
	Group x time	3.33	0.189	6.53	0.038*
BMI	Group effect	3.36	0.067	2.32	0.127
	Time effect	58.46	< 0.001*	65.26	< 0.001*
	Group x time	5.31	0.070	8.23	0.016*
Energy intake	Group effect	4.37	0.037*	3.90	0.048*
	Time effect	162.64	< 0.001*	187.45	< 0.001*
	Group x time	6.54	0.038*	10.33	0.006*
Protein intake	Group effect	1.75	0.185	1.24	0.265
	Time effect	96.41	< 0.001*	109.83	< 0.001*
	Group x time	4.85	0.088	7.38	0.025*
Compliance with energy requirement	Group effect	7.08	0.008*	4.88	0.027*
	Time effect	231.87	< 0.001*	280.13	< 0.001*
	Group x time	10.28	0.006*	14.48	0.001*
Compliance with protein requirement	Group effect	3.74	0.053	2.29	0.130
	Time effect	122.88	< 0.001*	151.55	< 0.001*
	Group x time	9.57	0.008*	13.78	0.001*
HAPA	Group effect	41.72	< 0.001*	34.81	< 0.001*
	Time effect	2.37	0.306	3.67	0.160
	Group x time	10.52	0.005*	10.04	0.007*
GSRS	Group effect	2.21	0.137	2.00	0.158
	Time effect	0.71	0.700	2.92	0.232
	Group x time	1.02	0.601	4.03	0.133
QoL	Group effect	3.49	0.062	4.95	0.056
	Time effect	2.47	0.292	4.18	0.124
	Group x time	0.73	0.695	0.54	0.764

Notes. GEE = Generalized estimating equations; ITT = Intention-to-treat; PG-SGA = Patient-Generated Subjective Global Assessment; NRS2002 = Nutritional risk screening 2002; HAPA Scale= Health Action Process Approach Theory Scale; GSRS = Gastrointestinal Symptom Rating Scale; QLQ-C30 = European Organization for the Research and Treatment of Cancer Quality of Life Questionnaire. $^{*}p<0.05$
