# Supplementary material for: Feasibility of an Individualized mHealth Nutrition (iNutrition) Intervention for Post-Discharged Gastric Cancer Patients Following Gastrectomy: A Randomized Controlled Pilot Trial
Source: Nutrients. 2023 Apr 13;15(8):1883. doi: 10.3390/nu15081883 (PMC10144256; doi:10.3390/nu15081883)
Supplement: Supplementary file 1 [file nutrients-15-01883-s001.zip › Supplementary material 2.pdf]

Supplementary Material 2: Qualitative analysis

Eleven patients exposed to the iNutrition intervention agreed to an end-of-trial interview. They were labeled P1 to P11 to protect their anonymity.

Risk perception

In this study, all the cases were postoperative gastric cancer patients who received a 12-week iNutrition intervention. Almost all participants (90.9%) were aware of the dangers associated with inadequate nutritional intake, which occurs due to anatomical changes, dietary alterations, and gastrointestinal symptoms following the major surgery. The participants mentioned some threats such as *weight loss* (P5, P7, & P10), *malnutrition* (P6, P10), *weakened immunity* (P1, P4), *anemia* (P2, P3), and *delayed chemotherapy due to low leucocyte amount* (P6, P9).

"*Two of my family members died of gastric cancer… I know that eating well is very important after the operation. If suffering from malnutrition, the subsequent treatment will be affected.*" (P6, Man, 54 years old)

"*I'm already thin as 40 kilos. If I'm going to get even thinner, how am I supposed to survive.*" (P7, Woman, 35 years old)

However, participants' experiences with losing weight were perceived differently, with a few who had previously been overweight describing such change as beneficial for their health, as P8 indicated:

"*I felt better than I had done for years because I used to be too heavy. and I had lost weight during the treatment.*" (P8, Man, 51years old)

Outcome expectancies

Patients engaged with the iNutrition intervention because they enjoyed the experience and believed it was doing them some good, increasing *knowledge* (P2, & P3), *nutritional intake* (P7, & P8), *giving them concrete plans to eat and cope with symptoms* (P4, & P7), and helping them to *gain weight* (P3, P5, & P7). They believed that if they fully adhere to the intervention, they would be better adapted to postoperative nutritional challenges. P2 hoped it would increase '*life expectancy.*'

"*The consultation and applet helped me a lot. I learned a lot of knowledge.*" (P2, Man, 49 years old)

"*One month after I was discharged from the hospital, I lost 2 kilos. And then I followed your advice and ate according to the weekly meal plans. Later, I gained the 2 kilos back, I think it's very effective.*" (P7, Woman, 50 years old)

For adherence to the iNutrition intervention, it was important that participants' perceived benefit might be not only an improvement in their physical status but also

psychological comfort, such as a perception of hope. For nine of the patients, the iNutrition intervention was enjoyable and restorative.

"*I was prepared mentally due to the knowledge of the program. I knew there might be some side effects during the early period after surgery which are common phenomena. And I knew how to eat if these side effects occurred. I think this made me more psychologically prepared to adapt to the challenge of eating.*" (P8, Man, 51 years old)

"I think for me the biggest improvement was just being able to gradually establish post-operative eating habits….I think it was a combination of the whole lot, the consultation and the applet, just the motivational aspects of the program of answering calls biweekly, using the applet freely….they weren't putting pressure on you to overdo things (P3, Woman, 71 years old)."

However, two patients revealed that the iNutrition intervention can have the unintended consequence of raising anxiety about malnutrition and disease.

"*I often would like to give up any therapy. It feels terrible... I know that it might be helpful to follow your nutrition plan. But I feel hopeless. I don't even want to go on with any therapy anymore.*" (P10, Man, 36 years old)

"*I can handle the situation about what to eat at home. I don't want to see anything about the terrible disease.*" (P11, Man, 66 years old)

Self-efficacy

Self-efficacy refers to if participants feel capable of engaging with the iNutrition intervention, how big they expect the effort to be when changing their eating habits according to the intervention, and how long they would persist in the face of obstacles. The majority of participants felt confident in their ability to adhere to the intervention. Two factors emerged as impacting self-efficacy: (1) personal factors; (2) support from family or carers.

I knew the importance of nutrition…I'm confident I will continue to do my best to achieve the nutrition requirement including energy and protein. (P6, Man, 50 years old)

My daughter is a nurse. She encouraged me to follow your plans. She also helped cook... We will kind of do whatever it takes. (P7, Woman, 50 years old)

However, family members could also be a reason for thinking the intervention is unimportant. '*My wife is an old-fashioned cook. She does everything right*'. The patient might also describe themselves as '*knowing about calories'* (P11, Man, 66 years old)

One participant noted that if the demands on their family members were to increase, their self-efficacy would decrease.

I'm not confident to adhere to the program myself. I can't cook myself. And my wife was busy with work and cooking for the family. She has no time to adhere to your advice. And I don't want to burden her with my demands...So I passively put limits on the times of meals and the intake. (P10, Man, 36 years old)

Intention

The majority of participants highlighted their intentions to engage with the iNutrition intervention. A participant "*hope it (iNutrition) can be used by more patients*" (P8). The factor influencing participants' intentions is if the intervention conflict with their existing knowledge or knowledge from a more trusting approach.

"I will refer to your instructions if they are correct. If I don't think they are correct, I won't follow them." (P1, Man, 47 years old)

Some participants who received advice from other sources believed that there are many food taboos and a link existed between cancer recurrence and diet.

"The doctor didn't say I can't have beef, chicken, and duck, but I haven't eaten any because [my wife] said so. She said these foods would promote cancer recurrence. I am already banned from eggs, beef, chicken, and duck." (P11, Man, 66 years old)

"My Chinese medicine doctor told me not to eat seafood (fish, shrimp, and so on). I need to take Chinese medicine to recuperate my body. And after drinking a large bowl of Chinese medicine, I'm already full, and couldn't eat more." (P9, Woman, 43 years old)

Barriers

Participants mentioned three barriers related to adhering to the iNutrition intervention: (1) no improvement in physical condition (such as weight), (2) gastrointestinal symptoms, and (3) imperfect applet.

I realized that I had to do something to maintain weight, I thought I would benefit from the program...My weight continued to drop….I thought it won't help me. (P6, Man, 50 years old)

After the beginning of chemotherapy, I got a feeling like hyperemesis gravidarum. And I'd hardly eaten anything in the following two weeks. (P4, Woman, 61 years old)

The iNutrition applet was user-friendly, and most participants could operate it with ease and independently. However, when participants with limited experience with technology were more apprehensive about their ability to take part in the program:

"When I heard it first, I thought I'm not au-fait with tech that much and I was kind of nervous." (P3, Woman, 71 years old)

Participants proposed several suggestions for the applet: The applet could be more *flexible and give more food choices* (P6). And *more information about new effective treatments (such as new drugs and curative effects)* is also needed (P2). The teaching videos in the applet were too long and lasted 3-5 minutes. *Dozens of seconds of video could be better*. (P2)

Resources

Participants mentioned the three resources of the iNutrition intervention were (1) the multidisciplinary support, (2) the biweekly nutrition consultation through phone calls, and (3) the evidence-based knowledge in the applet.

"My doctor and nurse were in the nutrition management team that made me trust this program. Sure enough, I was free to ask questions to them." (P3, Woman, 71 years old)

"Every phone call could help me solve some problems and put my mind at ease." (P4, Woman, 61 years old)

"... it was a bit haphazard, with me trying out various things to find out if I could eat this or that. And the "weekly meal plan" and "restrict and benefit foods" in the applet effectively solved the problem." (P7, Woman, 50 years old)

"I recorded every meal in the applet, it can calculate my intake automatically. And I will check whether I meet the goals, including energy and protein. If not, I will find something to eat; if so, I don't need to eat." (P8, Man, 51 years old)

Planning

The majority of participants reported that planning was key to achieving adequate nutrition intake. This involved action planning, such as deciding what they were going to eat, the amounts, and the preparation method. Almost all participants described how they rely on the nutrition consultation and iNutrition applet to make action planning to get adequate nutritional intake.

Biweekly after the phone call, you updated the weekly meal plan for me. I would write down the plan and display it on the fridge. (P6, Man, 50 years old)

I used the applet every morning when I woke up. I looked at it before I buy and cook food. And I will eat the amount of food and ONS you recommend to meet my nutrition goals. (P3, Woman, 71 years old)

Sometimes I follow the meal plans of the applet to cook, and sometimes I made my own plans according to my nutrition goals in the applet. (P7, Woman, 50 years old)

The mentioned types of action planning helped participants to feel in control, and especially short-term maintainers were explicit about the need for action planning, while long-term maintainers to a greater extent relied on habitual behaviour.

After about 2 months, I don't need to follow your diet plan. I have adapted to the new eating habits. (P1, Man, 47 years old)

Participants also described the importance of coping plans which helped them to manage any barriers that might have jeopardized their adequate nutrition intake. Participants mentioned a series of gastrointestinal symptoms such as stomachache, loss of appetite, trouble swallowing, nausea, diarrhea, and taste changes. Some participants reported the symptom management and nutrition consultation had helped them cope with the symptoms and barriers.

According to the applet, I eat frequent small meals throughout the day, instead of 3 big meals, and set an alarm to remind me to eat. It helped me overcome the loss of appetite and maintain weight. (P7, Woman, 50 years old)

Following your advice, I ate a small amount of food every hour to prevent nausea when undergoing chemotherapy. And after the chemotherapy, I regained my appetite and ate high-energy and high-protein foods. (P5, Man, 65 years old)
